# The Association between Dietary Intake of Antioxidants and Ocular Disease

**DOI:** 10.3390/diseases5010003

**Published:** 2017-01-30

**Authors:** Andrea Braakhuis, Ryan Raman, Ehsan Vaghefi

**Affiliations:** 1 Discipline of Nutrition, Faculty of Medical and Health Sciences, The University of Auckland, 85 Park Road Grafton, Auckland, 1142, New Zealand; rram154@aucklanduni.ac.nz; 2 Department of Optometry and Vision Science, Faculty of Medical and Health Science, The University of Auckland, 85 Park Road Grafton, Auckland, 1142, New Zealand; e.vaghefi@auckland.ac.nz

**Keywords:** ocular pathology, diet, nutrition, oxidants

## Abstract

To assess the association between dietary antioxidant intake and the incidence of the three major oxidative stress-related eye diseases, cataracts, glaucoma, and age-related macular degeneration, 78 cases from the University of Auckland Optometry and Vision Science clinic and 149 controls were recruited. Participants completed an antioxidant food-frequency questionnaire, analysed through multiple logistic regression. Protective associations were identified with higher consumption of fruit and vegetables (OR = 0.99; 95% CI: 0.98, 1.00; *p* = 0.004), vitamin C (OR = 0.63; 95% CI: 0.23, 1.03; *p* = 0.022), and β-carotene (OR = 0.56; 95% CI: 0.15, 0.98; *p* = 0.007). Meanwhile, harmful associations were observed with greater consumption of meat/nuts (OR = 1.03; 95% CI: 1.01, 1.05; *p* = 0.006) and cholesterol (OR = 1.09; 95% CI: 1.50, 2.46; *p* = 0.005). Diets rich in fruit and vegetables appear to be protective against cataracts, glaucoma, and age-related macular degeneration, while diets higher in meat and nuts may increase the risk of oxidative stress-related eye diseases. In addition, higher intakes of vitamin C and β-carotene from food, with reduction of dietary cholesterol intake, may be beneficial towards the outcome of oxidative stress-related eye diseases.

## 1. Introduction

Visual impairment and blindness are important issues in public health systems around the world. Visual impairment is defined as a corrected visual acuity of 6/18 or worse in the better-seeing eye, and is estimated to affect approximately 285 million individuals worldwide [1]. Blindness is believed to affect 39 million of the total visually impaired population, and is defined as a corrected visual acuity of 3/60 or worse in the better-seeing eye [2]. The global issue of visual impairment and blindness is felt in New Zealand with close to 125,000 New Zealanders aged 40 or over (6.1% of the population in that age group) facing a form of visual impairment [3]. Of this population, 12,000 New Zealanders suffer from blindness and 86% of these cases occur among individuals aged 70 or over. The number of people aged 40 and over suffering from visual impairment is expected to rise to 174,000 by 2020, and those suffering from blindness will rise to 18,300 [1,2].

There are three primary causes of visual impairment, including: cataracts (CAT), age-related macular degeneration (AMD), and glaucoma (GLA) [1]. These three ocular diseases induce damage to the eye through mechanisms of oxidative stress [3]. The pathogenesis of CAT is poorly understood; however, oxidative stress appears to aid the progression of age-related cataracts by facilitating damage to cellular components and accumulating advanced glycation end products (AGEs). AGEs induce conformational changes within the lens, causing increased opacity. With regards to AMD, patients with AMD exhibited a greater amount of oxidative modifications to proteins and DNA in the Bruch’s membrane, drusen, and retinal pigment epithelium compared with age-matched controls. Human retinal pigment epithelium cells exposed to a regimen of reactive oxygen species showed an increased concentration of drusen, which are associated with a poor AMD prognosis. Oxidative stress may have a role in the pathogenesis of GLA, through degradation of the trabecular network, or through direct damage to the retinol ganglion cells. Degradation of the trabecular meshwork results in increased intraocular pressure due to a build-up of aqueous humour. An in-depth discussion of the pathophysiology of oxidative stress and ocular disease (CAT, AMD and GLA) is beyond the scope of this publication, and is discussed elsewhere [3].

Antioxidants represent the first line of defence against oxidative stress and are obtained through the diet and produced internally [3]. In the eye, the major antioxidants that have a protective role are ascorbic acid, reduced glutathione, and superoxide dismutase-catalase [3]. Ascorbic acid is found in high concentrations in various areas that include the cornea, central corneal epithelium, lachrymal film, vitreous humour and aqueous humour [3], suggesting an important role in antioxidant protection in ocular health.

Early research by Cao and colleagues [4] suggested that the plasma antioxidant capacity can be increased with consumption of a diet rich in sources of antioxidants, such as fruit and vegetables. The Age-Related Eye Disease Study (AREDS 1 and 2) clearly demonstrated the ability of antioxidant therapy to reduce the progression of AMD by 25% [5]. Although diets rich in antioxidants have been identified in several studies to reduce the incidence of oxidative stress-related eye diseases, various antioxidant supplement studies provided conflicting findings [6,7,8,9]. In contrast, the intake of other food groups such as meat and related micronutrients such as cholesterol has been identified in various studies to increase the risk of oxidative stress-related eye conditions [10,11,12,13]. The impact of dietary antioxidant intake on the risk of oxidative stress-related eye diseases has been examined previously [13], but typically the studies addressed the effect of antioxidant supplements or fruit and vegetables in isolation, rather than the total dietary intake [14].

With regards to CAT, the evaluation of the literature indicates a higher dietary consumption of fruit and vegetables and vitamin C (including supplementation) appears to be associated with the most consistent risk reduction [13,14]. Vitamin E dietary intake and supplementation was also consistent with risk reduction in the majority of studies; however, findings on vitamin E supplementation suggested no observed differences in the rate of cataract extraction and lens characteristics between groups [5]. Higher dietary intakes of meat conversely appeared to increase the risk of CAT. With regards to vitamin A, findings by Theodoropoulou and colleagues [13] identified a harmful association (OR = 1.47) with higher intakes of retinol and a protective association with carotene (OR = 0.56). However, it may be possible that the harmful association identified with retinol may be related to dietary meat intake as retinol is the form of vitamin A found in animal products such as liver, kidneys, and eggs, and dietary meat intake was further identified in this study to increase the risk of CAT (OR 1.46).

Far less research has been conducted on AMD, with currently little evidence that dietary intake of fruit and vegetables or dietary antioxidants influence disease risk [3]. Human retinal pigment epithelium cells exposed to a regimen of reactive oxygen species showed an increased concentration of drusen, which are associated with a poor AMD prognosis [15,16]. The dietary intake of meat, in contrast, appeared to increase the risk of AMD when consumed frequently and in large quantities [12].

Oxidative stress is thought to degrade the trabecular network or cause damage to the retinol ganglion cells. Degradation of the trabecular meshwork results in increased intraocular pressure due to a build-up of aqueous humour [16]. However, due to the low number of published studies, the evidence regarding the prevention of GLA through higher consumption of antioxidant supplements and/or vegetables and fruit is currently unclear [3].

The majority of studies investigating the effect of diet on ocular disease incidence are conducted in European countries, and our aim was to study the association in a country that typically follows a Western diet.

## 2. Materials and Methods

A case-control study design was conducted to investigate the impact of dietary intake on the outcome of oxidative stress related eye-conditions: glaucoma, cataracts, and age-related macular degeneration within the New Zealand population. Participants with and without ocular disease were invited to complete a nutritional survey. Our study examined the association between the intake of specific food items, food groups, and micronutrients in the diet with ocular disease diagnosis.

Participants were sought from the University of Auckland Optometry and Vision science, and Hearing Tinnitus clinic database. Clients of the optometry clinic were designated as our sample case population, while clients of the hearing and tinnitus clinic were designated as our sample control population. Designation of hearing and tinnitus clients as our control population was as they were best matched for age, ethnicity, and population base.

Eligible participants were identified by clinic administrative staff by extending invites to clients aged 18 and over who had attended either clinic in the past 12–18 months. Optometry administrative staff further contacted clients who met the criteria above and asked for consent to be contacted by the researcher. A total of 588 eligible clients from the optometry clinic, and 522 from the hearing and tinnitus clinic were identified and contacted regarding the study. The researchers did not participate in the sampling process, to prevent coercion.

A total of 1110 letters were sent to identified participants comprised of: an invitation from the overall clinic manager, an invitation from the optometry department, participants information sheet, consent form, dietary intake survey including demographic questions, and a pre-paid return envelope. The research team collated the invitation packs for clinic staff to post. Participants were able to respond by sending completed forms through the pre-paid return envelope, or through an electronic version of the survey [17] with a link available on the information sheet.

A total of 280 responses were received after a one month period coinciding with the given deadline for submissions to be eligible to enter a prize draw. This is equivalent to a response rate of 25%. Of the 280 responses, 53 were excluded due to either: a lack of consent form, incomplete forms, or incorrectly completed forms. 227 responses were examined, and of this 149 participants self-reported as controls, while 78 participants self-reported as cases. Participants were classified as either a control or a case based on their response to the question “Do you currently, or have you ever required medical assistance for any eye related diseases?” Those responding with “no” were classified as controls and their validity was not further investigated. Those responding with “yes” were classified as cases and were further investigated by examining their clinical records and obtaining their visual acuity and ocular diagnosis. Those specifying yes but did not meet the case criteria were excluded. As a result a further 36 people were excluded, giving us a final case population of 42 people. Participant surveys and optometry files (if required) were recorded together and coded sequentially (i.e., the first completed participant will be coded as 1) to maintain anonymity.

Cases were any participant diagnosed with either: cataracts, age-related macular degeneration, primary open-angle glaucoma, or a combination of the three. Cases should have had no previous ocular surgery in both eyes (with the exception of cataract surgery). Although visual acuity score was obtained, it was not part of the exclusion criteria as removal of participants who did not meet the cut off would have impacted the associations derived from our research.

Controls were identified as participants with no prior medical conditions or treatments related to oxidative stress related eye conditions, or previous ocular surgeries. Controls were excluded if they had history of medical conditions or treatments known to be related to ocular conditions in the past. For the current study we chose to invite all clinic participants over a period of 12 to 18 months.

This study was approved by the University of Auckland Human Participants Ethics Committee on the 17th of February 2015 for three years, and has been performed to the ethical standards. Written informed consent was obtained from study participants.

### 2.1. Dietary Intake Questionnaire

All cases and controls completed a semi-quantitative food-frequency questionnaire; either by hand or electronically. The dietary questionnaire asked participants to indicate their average frequency of consumption of 31 food items or beverages per day, per week, per month, per year, and never. The questionnaire design was based on a prior study performed on the Australian population, examining the reliability of a 19-item food frequency questionnaire in assessing dietary antioxidant intake on health outcomes [18]. Meat and nuts are grouped together in the questionnaire, as major sources of protein. The questionnaire was modified alongside the New Zealand Adult Nutrition Survey, adding 13 more food items that were more commonly consumed in the New Zealand population [19]. The food items added/modified were green beans, oranges, mushrooms, potatoes, kumara, tea, coffee, white bread, wholemeal bread, alcohol, fish oil, multivitamins and other supplements. A pilot study was conducted on the final questionnaire of similar age to the clinic attendees for verification of comprehension of the New Zealand adapted version of the antioxidant questionnaire. Participants were correctly able to identify additional food and supplement items.

### 2.2. Data Entry

For purposes of statistical analysis, the frequency of consumption of different food items was quantified in terms of the number of times a food item was consumed per month. Therefore daily consumption was multiplied by 30, weekly consumption was multiplied by four, yearly consumption was divided by 12 and foods never consumed were given a value of 0. Food items were considered individually, and as groups as seen in other similar nutritional epidemiological [19]. Individual values for monthly consumption were added and the sums were approximately distributed into quintiles, based on the distribution of the entire study population. The food groups that were formed include; Meat and nuts, fruit and vegetables, dairy, breads and cereals, non-alcoholic beverages, alcohol, oil and added lipids, and supplements. Meat and nuts included: eggs, red meat, chicken, liver, kidney, nuts and seeds. Fruit and vegetables included: oranges, carrots, pumpkin, spinach, raw tomatoes, green beans, mushrooms, potatoes and kumara. Dairy included: skim or whole milk, cheese and yoghurt. Breads and cereals included: breakfast cereals, white bread and wholemeal bread. Non-alcoholic beverages included: tea and coffee. Alcohol as a group contained only alcohol. Oil and added lipids included: margarine and vegetable oils. Supplements included: fish oil, multivitamins, other vitamin supplements, and any other supplements. Specific items such as oil or added lipids were also considered individually due to the hypothesis of possible specific importance. Fats and lipids were labelled macronutrients, all remaining nutrients were labelled micronutrients including cholesterol.

Nutrient intakes for participants were estimated by multiplying the nutrient contents of the listed portion size for each specified food item by the frequency that the food item was consumed and summing the food items. The portion sizes specified were based off an earlier study in the Australian population examining the validity of a short food frequency questionnaire on ocular research [18]. The micronutrients examined in this analysis were retinol, β-carotene, vitamin C and vitamin E (α-tocopherol equivalents) as well as cholesterol. Values for the micronutrients per portion size were determined by the Foodworks (Xyris Ltd, 7th edition, Australia) dietary analysis software. Flavonoid intake was examined using data provided by the United States department of agriculture database for the flavonoid content of selected foods [20]. Flavonoid content from the classes: flavan-3-ols, flavones, flavonols, flavanones and anthocyanidins, were totaled for oranges, spinach and tea individually, and multiplied by their respective portion sizes and average monthly consumption per participant. Total flavonoid content was then determined by summating the three items. Oranges, spinach and tea were chosen as primary determinants of flavonoid content as they had the highest concentration of flavonoid per 100 mg.

### 2.3. Statistical Methods

Univariate analyses were initially performed to compare the frequency of intake distribution between the cases and controls by marginal quintiles of intake. We examined the association between dietary intake and glaucoma, age-related macular degeneration and cataracts by multiple logistic regression models. We controlled for several confounding factors, including age (ordinal in three age-groups as ≤59 years, 60–69 years, ≥70 years), sex (dichotomous, male vs female), secondary school qualifications (in eight groups as No secondary school qualifications, NZ School Certificate or National Certificate level 1 or NCEA level 1 or National Certificate level 1 or NCEA level 1, NZ Sixth Form Certificate or National Certificate level 2 or NZ UE before 1986 or NCEA level 2, NZ Higher School Certificate or NZ Higher School Certificate or NZ University NZ Higher School Certificate or NZ University NZ Higher School Certificate or NZ University Entrance from NZ Bursary or National Certificate level 3, NCEA (National Certificate of Educational Achievement) level 4, other NZ Secondary school qualification (specified), overseas secondary school qualification, don’t know, I do not wish to answer this question), body mass index (BMI) (ordinal in four categories as under-weight as ≤18.5, normal weight as between 18.5–25, overweight as ≥25–30, obese as ≥30), smoking habits and duration of smoking (as ex-smoker (yes or no) and pack-years among ex-smokers, current smoker (yes vs. no) and past years (among current smokers). The analysis was carried out taking glaucoma, cataracts and age-related macular degeneration as a general outcome, and then, alternatively, by glaucoma, cataracts, and age-related macular degeneration individually to explore whether the associations were specific to the type of oxidative stress-related ocular condition. All analyses were conducted using the MATLAB statistical toolbox (MathWorks, version R2016b, Portola Valley, CA, USA).

## 3. Results

### 3.1. Sample Population and Characteristics

The most frequent type of ocular condition was cataracts (*n* = 33), followed by a combination of the three (cataracts, glaucoma, and age-related macular degeneration) (*n* = 7), then age-related macular degeneration (*n* = 1), and glaucoma (*n* = 1). Participants with a combination of the three were comprised of cataracts and age-related macular degeneration (*n* = 4), and cataracts and glaucoma (*n* = 3). The distribution of disease type was skewed in favour of cataract-diagnosed participants as the major determinants of our population. Details on the demographics of our sample population are presented in Table 1. Mean characteristics were similar between the case and control populations, albeit the controls were slightly younger and smoked less.

### 3.2. Sample Population Dietary Intake by Groups

Table 2 shows the distribution of cases and controls by marginal quintiles of monthly frequency of consumption of eight food groups. A significant variation in mean intake was observed between the case and control groups for meat and nuts, fruit and vegetables, dairy and oils and added lipids. Variation in the mean intake was also observed between the case and control groups for alcohol, non-alcoholic beverages and supplements; however, the difference detected for these food groups was not statistically significant (see Table 2). There was no difference detected for the mean intake of breads and cereals between the case and control populations.

### 3.3. Sample Population Dietary Intake by Micronutrients

Table 3 shows the distribution of cases and controls by marginal quintiles of daily intake of micronutrients. A significant variation in the mean intake was observed between the case and control groups for cholesterol, vitamin C, and β-carotene. There was no variation detected between the case and control populations for the mean intake of vitamin E and the total flavonoid intake (see Table 3).

### 3.4. Multiple Logistic Regression Derived Odds Ratios for Food Groups

In Table 4, multiple logistic regression–derived odds ratios (ORs) and 95% confidence intervals (CI) with associated *p*-values per one standard deviation increment of the major categories for all ocular conditions (combined) are given. Significant inverse associations were observed with the consumption of fruit and vegetables. In contrast, the consumption of meat and nuts was significantly positively associated with all types of ocular diseases (see Table 4). No significant associations were observed between the outcome of all ocular conditions and the intakes of dairy, breads and cereals, non-alcoholic beverages, alcohol, oils and lipids, and supplements.

### 3.5. Multiple Logistic Regression-Derived Odds Ratios for Micronutrients

Table 5 presents multiple logistic regression–derived ORs for the association between all ocular diseases and micronutrients. There were significant negative associations between the intake of vitamin C, β-carotene and all types of ocular diseases. In contrast, there was a significant positive association between the intake of cholesterol and risk of all ocular disease (see Table 5). Positive associations were also observed between total flavonoid intake and vitamin E with the outcome of combined ocular diseases; however, these associations were not statistically significant.

## 4. Discussion

The current research aimed to investigate the relationship between the dietary intake of antioxidants from various food groups with the outcome of the oxidative stress-related eye diseases glaucoma, cataracts, and age-related macular degeneration. After adjusting for certain potential confounders, age, gender, BMI, education level and smoking habit, we identified that a higher intake of fruit and vegetables was associated with a reduced risk of oxidative stress-related eye diseases. In contrast, our findings identified a high consumption of meat and nuts was associated with an increased risk of oxidative stress-related ocular diseases. Of the micronutrients examined, our findings identified a higher intake of vitamin C and β-carotene was associated with a reduced risk of oxidative stress-related eye diseases. In contrast, our findings identified a higher intake of cholesterol was associated with an increased risk of oxidative stress-related eye diseases.

In the sample population of cases, 79% were diagnosed with cataracts only, 9.5% were diagnosed with a combination of cataracts and age-related macular degeneration, 7.1% were diagnosed with a combination of cataracts and glaucoma, 2.4% were diagnosed with age-related macular degeneration only, and 2.4% were diagnosed with glaucoma only. The distribution of our participants is skewed in favour of participants with cataracts; therefore, our results are best applied to a population with a similar distribution of oxidative stress-related eye diseases. We compared the distribution of oxidative stress-related eye diseases in our sample population with the distribution of oxidative stress-related eye diseases in New Zealand to determine the extent of the similarity. Figures derived from the Clear Focus report [21] show that within the New Zealand population, 55% of vision loss is related to uncorrected refractive error, 13% of vision loss is related to cataracts, 9% of vision loss cases are related to age-related macular degeneration, and 4% of vision loss cases are related to glaucoma [21]. Although cataracts appear to contribute to the greatest proportion of the visually impaired population within New Zealand compared to the other oxidative stress-related eye diseases, our distribution of participants diagnosed with cataracts compared to other ocular conditions was still skewed in their favour. This may affect the validity of direct recommendations made from our research.

Our finding of higher fruit and vegetable intake being associated with a reduced risk of oxidative stress-related eye diseases is consistent with findings from previous studies [3,13,22]. The protective quality identified between higher intakes of fruit and vegetables with a reduced risk of oxidative stress-related eye diseases may be attributed to their composition as they are rich sources of dietary antioxidants. Vegetables such as spinach contain lutein and zeaxanthin and have been shown to improve visual acuity and retinal function in those with early AMD, and may show promise with CAT and GLA [23]. Dietary antioxidants have a key role in oxidative stress reduction by inhibiting oxidative reactions and removing free radical intermediates [24]. Our findings of higher dietary intake of vitamin C and reduced outcomes of glaucoma, cataracts, and age-related macular degeneration are consistent with findings from previous studies [25,26,27,28,29]. β-carotene was also identified to reduce the risk of oxidative stress-related eye diseases with higher dietary intake. Our finding of higher dietary intake of beta carotene being associated with a reduced risk of oxidative stress-related eye diseases is consistent with previous research [13,30,31,32,33,34].

Interestingly, trivial or unclear associations were found between the consumption of flavonoids and the incidence of ocular disease, in contrast to emerging data on the effect of flavonoid consumption on visual acuity in those with GLA [29]. The dietary consumption of flavonoids in the study population ranged from 0.15 to 2 g daily, which is similar to the values reported in the general population [3]. The range is quite varied and studies demonstrating a benefit to ocular function generally supplement with flavonoids, rather than rely on dietary intake. The bioavailability of flavonoids is quite low, potentially affecting outcomes [29].

In contrast, our research identified a harmful association between the dietary intake of meat and nuts with the outcome of oxidative stress-related eye diseases. Evidence suggests higher intakes of red meat are associated with elevated markers of oxidative stress [35,36]. Our findings are consistent with previous research in this area [10,11,12,13]; however, there is minimal evidence for an impact of dietary meat intake on glaucoma [11]. In the future, meat and nuts should be analysed separately, as, despite being major protein sources, they are quite different with regards to antioxidant content. Higher dietary intakes of cholesterol appeared to increase the risk of oxidative stress-related eye diseases in our study, and this is consistent with findings in the area of cataracts [10,11,13] and age-related macular degeneration [12]. Similar to dietary meat intake, there is currently no association identified between dietary cholesterol intake and glaucoma. It is uncertain why cholesterol might influence ocular disease incidence, and perhaps it is a function of the saturated fat often co-existing with high-cholesterol foods that may be of greater impact.

Our study has several strengths. Our research questionnaire was validated in previous ocular research on a similar population, and was tailored to meet the diets of the New Zealand population based on findings by the Ministry of Health [18,19]. This would provide us with dietary information best representing the New Zealand population. Our research questionnaire was available in both physical and electronic versions to encourage greater response rates. The ease and cost of the research design were strengths of the study as a short questionnaire provided us with a greater response rate at a cheaper cost than a more expansive and detailed questionnaire would have provided.

Several limitations were identified with our research. Participants who self-identified as controls were not investigated for their validity. Therefore, there may have been controls with oxidative stress-related eye diseases, latent ocular conditions, or other ocular impairments. Our questionnaire only assessed the intake of 31 food items, whereas similar research by [13] assessed the dietary intake of 120 food items. Our assessment criteria of those diagnosed with pre-existing ocular disease will fail to recruit early disease status, at which point the diet may have the strongest influence. Future research could focus on the associations dietary antioxidants have with disease severity and incidence.

## 5. Conclusions

The findings presented from our research support the role of nutritional processes in the outcomes of oxidative stress-related eye diseases, in particular cataracts, glaucoma, and age-related macular degeneration. From the food groups assessed, our research suggests a protective association with higher intakes of fruits and vegetables, and a harmful association with higher intakes of meat and nuts. From the micronutrients assessed, our research was able to identify protective associations with higher intakes of vitamin C and β-carotene, while higher intakes of cholesterol presented a harmful association. Dietary advice along these lines may contribute towards public health guidelines for a reduction in the risk of cataracts, glaucoma, and age-related macular degeneration, in a population with a similar distribution of cases as our study.

## Figures and Tables

**Table 1:** Demographic data from sample populations of 42 cases with glaucoma, cataracts, age-related macular degeneration, or a combination of the three, and 149 controls (mean ± standard deviation (SD)).

Characteristic	Case	Control
Age (years)	74.8 (±12.1)	64.7 (±14.0)
Height (cm)	167.6 (±10.5)	169.6 (±9.6)
Weight (kg)	71.1 (±15.2)	73.4 (±15.1)
BMI (kg/m^2^)	24.6 (±4.4)	25.0 (±5.3)
Cigarettes Smoked Per Day	7.1 (±15.0)	4.8 (±8.8)
Smoking Years	10.9 (±16.0)	6.3 (±12.0)

**Table 2:** Distribution of 42 cases with either glaucoma, age-related macular degeneration, cataracts, or a combination of the three, and 149 control subjects, by monthly frequency of consumption of various food groups. The quintiles spread of participants are shown in Q1 to Q5.

Food Group	Q1 (Lowest)	Q2	Q3	Q4	Q5 (Highest)	*F-*value	*p*-value
Meat and Nuts						11.467	<0.001
Case	5	23	31	13	6		
Control	20	64	50	8	3		
Quintile median (times/month)	9	28	41	58	69.5		
Fruit and vegetables						12.276	<0.001
Case	9	31	26	11	1		
Control	16	44	44	29	12		
Quintile median (times/month)	29	54.5	86.2	123	163		
Dairy						4.479	0.036
Case	9	25	21	17	6		
Control	28	42	51	21	3		
Quintile median (times/month)	8.08	37	60.1	90	120		
Breads and Cereals						0.967	0.327
Case	9	35	21	9	4		
Control	30	68	33	9	5		
Quintile median (times/month)	24	60	90	120	150		
Non-Alcoholic Beverages						1.769	0.185
Case	8	20	28	12	11		
Control	32	38	49	15	11		
Quintile median (times/month)	30	60	96	150	197		
Alcohol						1.242	0.267
Case	46	12	20	1	3		
Control	86	36	28	2	7		
Oils and added lipids						5.89	0.016
Case	16	28	19	5	10		
Control	35	70	30	6	4		
Quintile median (times/month)	8	34	60	90	120		
Supplements						1.489	0.224
Case	40	19	8	8	4		
Control	86	38	16	3	2		
Quintile median (times/month)	0	31	60	90	117		

**Table 3:** Distribution of 42 cases with either glaucoma, age-related macular degeneration, cataracts, or a combination of the three, and 149 control subjects, by marginal quintiles of daily intake of micronutrients.

Nutrient	Q1 (Lowest)	Q2	Q3	Q4	Q5 (Highest)	*F-*value	*p*-value
Cholesterol (mg)						12.030	0.005
Case	3	23	30	15	7		
Control	17	60	56	9	3		
Quintile median (mg/day)	286	896	39980	57015	68888		
Vitamin C (mg)						6.293	0.022
Case	8	26	28	15	1		
Control	12	44	48	29	12		
Quintile median (mg/day)	156	278	442	579	761		
Vitamin E (mg)						0.388	0.830
Case	14	41	14	4	5		
Control	38	74	20	6	7		
Quintile median (mg/day)	20.3	30.0	41.6	53.4	69.4		
B-carotene (µg)						10.263	0.007
Case	9	31	26	11	1		
Control	15	44	45	27	14		
Quintile median (µg/day)	7521	13620	21175	29269	38727		
Total Flavonoid (mg)						0.003	0.463
Case	30	27	9	9	3		
Control	63	42	19	16	5		
Quintile median (mg/day)	120	541	888	1184	1486		

**Table 4:** Multiple logistic regression derived ORs and 95% CI with associated *p*-values per one standard deviation increment of the major categories of food groups for all ocular conditions combined.

Food Group	OR	CI (95%)-Low	CI (95%)-High	*p*-value
Meat and Nuts	1.03	1.01	1.05	0.006
Fruit and veg	0.99	0.98	1.00	0.004
Dairy	1.01	0.99	1.02	0.352
Breads and Cereals	1.01	1.00	1.02	0.114
Non-Alcoholic Beverages	1.00	1.00	1.01	0.339
Alcohol	0.98	0.97	1.00	0.074
Oil and added lipids	1.01	1.00	1.02	0.059
Supplements	1.00	0.99	1.01	0.686

**Table 5:** Multiple logistic regression derived ORs and 95% CI with associated *p*-values per one standard deviation increment of the major categories of micronutrients for all ocular conditions combined.

Nutrient	OR	CI (95%)-Low	CI (95%)-High	*p*-value
Cholesterol (mg)	1.98	1.50	2.46	0.005
Vitamin C (mg)	0.63	0.23	1.03	0.022
Vitamin E (mg)	1.04	0.67	1.41	0.830
B-carotene (µg)	0.56	0.15	0.98	0.007
Total Flavonoid (mg)	1.14	0.80	1.48	0.463
